# The prognostic value of gray-white-matter ratio in cardiac arrest patients treated with hypothermia

**DOI:** 10.1186/1757-7241-21-23

**Published:** 2013-04-08

**Authors:** Michael Scheel, Christian Storm, Andre Gentsch, Jens Nee, Fridolin Luckenbach, Christoph J Ploner, Christoph Leithner

**Affiliations:** 1 Department of Neuroradiology, Charité-Universitätsmedizin Berlin, Berlin, Germany; 2 Department of Neurology, Charité-Universitätsmedizin Berlin, Berlin, Germany; 3 Department of Intensive Care Medicine and Nephrology, Charité-Universitätsmedizin Berlin, Berlin, Germany; 4 Department of Neuroradiology, Charité-Universitätsmedizin Berlin, Charitéplatz 1, Berlin, 10117, Germany

**Keywords:** Hypoxic ischemic encephalopathy, Hypothermia, Cardiac arrest, Prognosis, Computed tomography, Gray-white matter ratio

## Abstract

**Background:**

Mild therapeutic hypothermia alters the validity of a number of parameters currently used to predict neurological outcome after cardiac arrest and resuscitation. Thus, additional parameters are needed to increase certainty of early prognosis in these patients. A promising new approach is the determination of the gray-white-matter ratio (GWR) in cranial computed tomography (CCT) obtained early after resuscitation. It is not known how GWR relates to established outcome parameters such as neuron specific enolase (NSE) or somatosensory evoked potentials (SSEP).

**Methods:**

Cardiac arrest patients (n = 98) treated with hypothermia were retrospectively analyzed with respect to the prognostic value of GWR, NSE and SSEP.

**Results:**

A GWR < 1.16 predicted poor outcome with 100% specificity and 38% sensitivity. In 62 patients NSE, SSEP and CCT were available. The sensitivity of poor outcome prediction by both NSE > 97 μg/L and bilateral absent SSEP was 43%. The sensitivity increased to 53% in a multi-parameter approach predicting poor outcome using at least two of the three parameters (GWR, NSE and SSEP).

**Conclusion:**

Our results suggest a strong association of a low GWR with poor outcome following cardiac arrest. Determination of the GWR increases the sensitivity in a multi-parameter approach for prediction of poor outcome after cardiac arrest.

## Introduction

An increasing number of recent studies on outcome prognostication after cardiac arrest demonstrate that hypothermia treatment alters prognostic parameters that had been established in normothermic patients. For example, a level of neuron specific enolase (NSE) above 33 μg/L, obtained on day one to three after cardiac arrest, and motor reaction to painful stimuli not better than extension on day three, are associated with a substantial rate of false poor outcome predictions in these patients [1-8]. Individual patients have been reported who survived with good neurological outcome despite NSE levels of 97 μg/L [3], bilateral absent N20 of medianus somatosensory evoked potentials (SSEP) [4,5], absent pupillary light responses or corneal reflexes [6,7] and early status epilepticus [8]. Before limitation of treatment is considered, a high level of certainty is needed and the combination of several prognostic parameters may increase the level of certainty for poor outcome prediction.

Brain imaging appears a self-evident choice for prediction of neurological outcome in patients with suspected hypoxic-ischemic encephalopathy. Severe cases are characterized by cerebral edema with decreased attenuation of gray matter in cranial computed tomography (CCT). A loss of contrast between gray and white matter has been shown to be associated with poor outcome [9,10]. Recent studies have evaluated CT signs, such as cerebral gray-white matter ratio (GWR) as an early prognostic outcome parameter in hypoxic-ischemic encephalopathy [11-16]. GWR is obtained by measuring Hounsfield units (HU) in gray and white matter and calculating the ratio of both [17]. In severe cases of cerebral edema, the ratio decreases to values below unity, also known as the ‘reversal sign’ [18].

There is currently no consensus on a distinct GWR cut-off value that may predict poor outcome with high specificity. A small number of studies on this subject suggest values between 1.15 [17] and 1.22 [13], respectively. Furthermore, it is neither known whether hypothermia affects the prognostic significance of a reduced GWR, nor whether the sensitivity with which poor outcome is predicted by NSE and SSEP can be increased by measuring the GWR. We therefore retrospectively investigated the prognostic value of GWR in a cohort of cardiac arrest patients treated with hypothermia and compared GWR with the established outcome parameters NSE and SSEP.

## Materials and methods

### Subjects

A total number of 353 consecutive patients after cardiac arrest were prospectively included into our database between 12/2005 and 10/2011. Since 2005, all patients have been treated with mild hypothermia in accordance with established guidelines (details of this treatment have been previously described [2]). The study protocol was approved by the local ethics committee on human research (Charité - Ethikkommission) and was conducted in accordance with the guidelines of the Declaration of Helsinki. Written informed consent to the use of routine clinical data is part of the standard contract between patients and the University Hospital Charité Berlin and was obtained from patients or their legal representatives. All data were collected from routine investigations in patients resuscitated from cardiac arrest. In our department, neurological assessment of these patients comprises several clinical, laboratory and electrophysiological investigations. Treatment decisions are always based on combined results from these examinations. Except for patients with confirmed brain death, treatment is continued for at least seven days in all patients [19].

In our database, we identified a subgroup of patients (n = 111) that had received cranial computed tomography (CCT) within the first seven days after cardiac arrest (median = 5 h; IQR = 2–24 hours). CCT was ordered independently of this study by the treating physician either to rule out a primary intracranial event leading to cardiac arrest (e.g. subarachnoid hemorrhage) or when clinical findings suggested intracranial complications (e.g. intracranial hemorrhage during anticoagulant therapy for myocardial infarction). Thirteen patients were excluded from further analysis for findings that would have biased measurement of Hounsfield units and GWR calculation (only contrast enhanced CCT available (3), hydrocephalus and shunt artifact (3), severe movement artifacts (2), intracerebral hemorrhage (3), old large ischemic lesion (1), massive calcification of the basal ganglia (1)).

Ninety-eight patients were included in the final analysis. All patients were treated with mild hypothermia. Table 1 summarizes the demographic data. Outcome was determined using the cerebral performance category (CPC) score [20], obtained by the treating physician at discharge from the intensive care unit (ICU). Good outcome was defined as CPC 1–2 and poor outcome as CPC 3–5.

**Table 1:** Demographic data given as absolute numbers and percent or median and interquartile range (IQR)

**Demographics of study population**	
Age (years)	61 (47–72.2)
Female sex	33 (33.7%)
APACHE Score	30 (23.5–36)
Out-of-hospital	81 (82.7%)
*Primary cause of arrest*	
Cardiac	52 (53%)
Respiratory	41 (42%)
Other	5 (5%)
Shockable rhythm	29 (29.6%)
Time to ROSC (min)	14.5 (10–26)
Total epinephrine dose (mg)	2 (0.7–5.0)
Length of ICU stay (days)	11.5 (5–28)
Time on ventilator (hours)	205 (109–521.2)
*Neurological outcome*	
CPC 1	23 (23.5%)
CPC 2	14 (14.3%)
CPC 3	2 (2.0%)
CPC 4	9 (9.2%)
CPC 5	50 (51%)

APACHE: Acute Physiology and Chronic Health Evaluation, AMI: acute myocardial infarction, ROSC: return of spontaneous circulation, ICU: intensive care unit, CPC: cerebral performance category.

### CCT acquisition, region of interest (ROI) measurements and GWR calculation

Only non-contrast enhanced CT scans were analyzed. CT scans were acquired on three different GE scanners (GE LightSpeed Pro 16 (n = 78), Lightspeed Ultra (n = 7), Lightspeed VCT (n = 13)-GE Healthcare, Little Chalfont, UK). All examinations followed a standard head CT protocol with a slice thickness of 5 mm.

For assessment of inter-raterreliability, ROIs were determined independently by two raters. ROIs were placed and GWR scores calculated according to criteria described previously [17]. The ROIs consisted of circular shaped areas (0.1 cm^2^) placed bilaterally in caudate nucleus (CN), putamen (PU), thalamus (THL), posterior limb of internal capsule (PIC), forceps minor of the corpus callosum (CC), medial cortex (MC1) and medial white matter (MWM1) at the level of the centrum semiovale and high convexity area (MC2 and MWM2, respectively). Both raters were blind to clinical information and outcome of all patients as well as ROI placement and GWR scores of each other. GWR in the basal ganglia was calculated as GWR-BG = (CN + PU) / (CC + PIC). GWR at the cortical level was calculated as GWR-CE = (MC1 + MC2) / (MWM1 + MWM2). Average GWR was defined as the average of these two GWR-AV = (GWR-BG + GWR-CE)/2 [17].

### Neuron specific enolase and somatosensory evoked potentials

NSE serum levels were determined in 84 out of 98 patients as described in an earlier study [2]. We have previously found a cut-off value of 78.9 μg/L for poor outcome prediction with 100% specificity [2], and a cut-off value of 81.8 μg/L has recently been reported by a large prospective multicenter study [6]. To our knowledge, the highest NSE cut-off value reported is 97 μg/L [3]. We therefore applied this limit to the current study and have implemented it in our diagnostic pathway [19].

For 67 out of 98 patients results of SSEP were available. SSEPs were performed with stimulation of the median nerve at the wrist as described earlier [4]. Results were classified as bilateral absent N20 or at least unilateral present. The majority of SSEPs were performed between 48 and 96 hours after cardiac arrest.

In 62 out of 98 patients, all three parameters (GWR, NSE and SSEP) were available. In this patient cohort, we determined sensitivity and specificity for poor outcome prediction for all three parameters separately as well as for the combination of SSEP and NSE (predicting poor outcome if both NSE > 97 μg/L and bilateral absent N20) and for the combination of SSEP, NSE and GWR (predicting poor outcome by at least two of three parameters, NSE > 97 μg/L, bilateral absent N20, GWR < 1.16).

### Statistical analysis

We calculated the correlation coefficients for attenuation measurements determined by two different raters (MS and AG) for GWR measures to assess inter-rater reliability. Absolute attenuation values and GWR values were compared between patients with good outcome (CPC 1–2) and poor outcome (CPC 3–5) by means of a Mann-Whitney-U test. The central aim of the study was to determine whether a cut-off level of GWR exists that allows prediction of poor outcome with specificity close to 100%. Additionally, we conducted ROC analyses for the three different GWR measurements (GWR-BG, GWR-CE, GWR-AV) to illustrate the overall performance of GWR in discrimination of patients with good and poor outcome.

To evaluate a possible relationship of GWR with time between cardiac arrest and CCT, we divided all patients with poor outcome into three groups (CCT <6 h, 6–24 h, >24 h after cardiac arrest). GWR values of these three groups were compared by using a Kruskal-Wallis test.

## Results

### Association of GWR and clinical outcome

**Figure 1:**
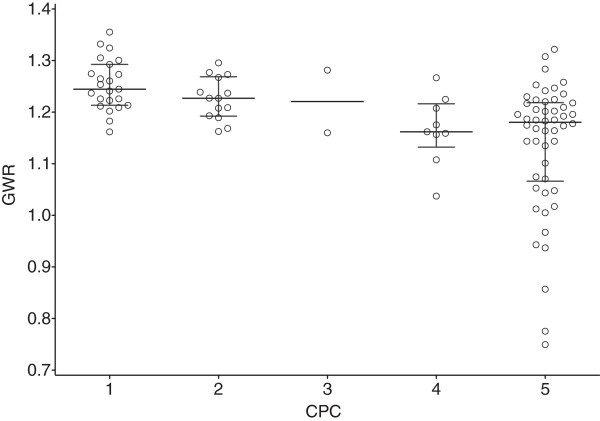
**Association of GWR with outcome.** Median and interquartile range are shown. Circles denote individual patients. No patient with GWR < 1.16 had CPC 1–2 while 38% of patients with CPC 3–5 had GWR < 1.16.

Inter-rater reliability of GWR calculation was excellent with an overall Pearson’s correlation coefficient of 0.97 determined from all measurements combined. Similar to previous studies, we found a highly significant association between GWR and clinical outcome (Figure 1 and Table 2). Absolute attenuation values in all gray matter regions (CN, PU, THL, MC1, MC2) were significantly lower in patients with poor outcome. In contrast, the absolute attenuation in white matter regions (CC, PIC, MWM1, MWM2) revealed no significant difference (Table 2).

**Table 2:** Absolute attenuation in Hounsfield Units and GWR calculations

	**Good outcome**	**Poor outcome**	**p-value**
	**(CPC 1–2)**	**(CPC 3–5)**	**MWU test**
	**n = 37**	**n = 61**	
**CN**	33.7 (32.5–35.1)	31.3 (28.1–33.2)	< 0.001
**PU**	34.7 (33.7–36.1)	32.8 (30.1–34.7)	< 0.001
**THL**	32.9 (31.2–34.3)	31.1 (29.5–32.8)	0.002
**CC**	27.5 (26.6–28.3)	26.7 (25.7–28.7)	0.241
**PIC**	27.4 (26.2–28.5)	27.7 (26.5–29.0)	0.459
**MC1**	31.6 (30.8–32.7)	29.5 (28.2–32.3)	< 0.001
**MC2**	31.5 (30.3–32.2)	29.4 (27.5–30.7)	< 0.001
**MWM1**	25.9 (24.1–27.2)	25.8 (24.3–27.3)	0.860
**MWM2**	25.9 (24.3–26.7)	26.0 (24.3–27.3)	0.514
**GWR-AV**	1.24 (1.21–1.27)	1.18 (1.10–1.22)	< 0.001
**GWR-BG**	1.26 (1.23–1.29)	1.18 (1.10–1.25)	< 0.001
**GWR-CE**	1.22 (1.17–1.28)	1.17 (1.10–1.20)	< 0.001

The table shows Median (IQR) for each group and the respective p-value of the Mann-Whitney-Utest. Caudate nucleus-CN, putamen-PU, thalamus-THL, CC-corpus callosum, posterior limb of internal capsule-PIC, MC1 and MWM1-medial cortex and medial white matter at the level of the centrum semiovale, MC2 and MWM2-medial cortex and medial white matter at the level of the high convexity area.

**Table 3:** Single parameter approach-sensitivity and negative predictive values (at 100% specificity) with respect to poor outcome prediction

	**No. of patients**	**CPC 3–5**	**CPC 1–2**	**Sensitivity**	**NPV**
		**‘poor’**	**‘good’**		
**GWR**_**AV**_ **< 1.16**	98	23	0	37.7%	49.3%
**GWR**_**AV**_ **≥ 1.16**		38	37		
**NSE > 97 μg/L**	84	26	0	53.1%	60.3%
**NSE ≤ 97 μg/L**		23	35		
**SSEP absent**	67	25	0	56.8%	54.8%
**SSEP detected**		19	23		

ROC analysis demonstrated an area under the curve (AUC) for GWR-AV = 0.81, GWR-BG = 0.77 and GWR-CE = 0.75 (Additional file 1: Figure S1). Therefore we used GWR-AV for all subsequent analysis and refer to it as GWR in the following.

GWR < 1.16 predicted poor outcome with a sensitivity of 38% and a specificity of 100%. Applying a GWR threshold of 1.2 would have misclassified 6 patients (CPC 1/2 = 2/4 patients).

**Figure 2:**
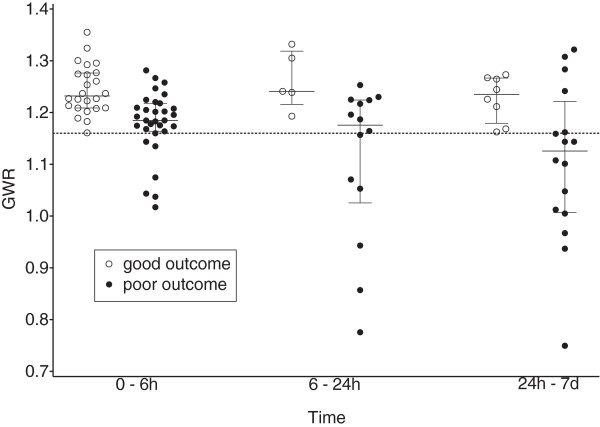
**GWR time dependency with respect to outcome.** GWR median and interquartile range are shown for different time points. Circles denote individual patients. CPC 1–2 was defined as good outcome, CPC 3–5 as poor outcome.

Median GWR values in patients with poor outcome were higher when CCT was performed early, i.e. within the first 6 hours (median = 1.18, IQR = 1.16–1.21) compared to examinations performed between 6–24 hours (median = 1.17, IQR = 1.03–1.22) and after 24 hours (median = 1.13, IQR = 1.01–1.22) (Figure 2). These differences however were not statistically significant (Kruskal-Wallis test = 4.02; p = 0.13).

### Comparison of GWR, NSE and SSEP

Table 3 gives an overview on sensitivity and negative predictive value of GWR, NSE and SSEP for the whole study cohort. In our study, 62 Patients had received CCT, NSE and SSEP. In this subgroup, poor outcome was detected with a sensitivity of 53% by NSE > 97 μg/L, 55% by bilaterally absent SSEP and 35% by GWR < 1.16. No patient with bilateral absent SSEP, NSE > 97 μg/L or GWR < 1.16 survived with good outcome. NSE and SSEP detected poor outcome for only partly overlapping patient populations: 65% of patients (26/40) with poor outcome had absent SSEP and/or NSE > 97 μg/L and 70% of patients (28/40) with poor outcome had at least one of three parameters (SSEP, NSE, GWR) suggesting poor outcome. Hence, GWR only marginally increased sensitivity for poor outcome detection in a prediction approach that requires only one positive parameter.

Predicting poor outcome if both, NSE > 97 μg/L and SSEP bilaterally absent, yielded a sensitivity of 43%. With this approach (at least two parameters pathologic for prediction of poor outcome), GWR increased sensitivity to 53%. Table 4 gives an overview of prediction by NSE, SSEP and GWR for the subgroup of patients (n = 62) for which all three parameters were available.

**Table 4:** Single and multi parameter approach-sensitivity and negative predictive values (at 100% specificity) for poor outcome prediction in the subgroup of 62 patients for which NSE, SSEP and CCT were available

	**No. of patients**	**CPC 3–5**	**CPC 1–2**	**Sensitivity**	**NPV**
		**‘poor’**	**‘good’**		
**GWR**_**AV**_ **< 1.16**	62	14	0	35.0%	45.8%
**GWR**_**AV**_ **≥ 1.16**		26	22		
**NSE > 97 μg/L**	62	21	0	52.5%	53.7%
**NSE ≤ 97 μg/L**		19	22		
**SSEP absent**	62	22	0	55.0%	55.0%
**SSEP detected**		18	22		
**SSEP absent and/or NSE > 97 μg/L**	62	26	0	65.0%	61.0%
**SSEP detected and NSE < 97 μg/L**		14	22		
**SSEP absent and NSE > 97 μg/L**	62	17	0	42.5%	48.9%
**SSEP detected or NSE < 97 μg/L**		23	22		
**2 or 3 abnormal (SSEP, NSE, GWR)**	62	21	0	52.5%	53.7%
**1 or 0 abnormal (SSEP, NSE, GWR)**		19	22		
**One or more parameters pathologic**	62	28	0	70.0%	64.7%
**All parameters normal**		12	22		

## Discussion

Our study suggests that a gray-whitematter ratio (GWR) below 1.16 may be a specific parameter for prediction of poor outcome in patients treated with hypothermia. This corroborates previous findings that were obtained in normothermia and mixed patient cohorts (normothermia and hypothermia). None of 23 patients with a GWR < 1.16 survived with good outcome.

The majority of patients with GWR < 1.16 had at least one other parameter indicative of poor outcome (NSE > 97 μg/L and/or bilateral absent SSEP). Therefore, GWR did not substantially increase the sensitivity for poor outcome detection, if one accepts poor outcome prediction by a single parameter. However, because individual patients may survive despite poor outcome prediction by single parameters, it has been argued that poor outcome prediction should be based on a multi-parameter approach [6]. We demonstrated in a subgroup of 62 patients who had received NSE, SSEP and CCT that a GWR < 1.16 increased the sensitivity of poor outcome detection (from 43% (17/40) to 53% (n = 21/40)) if at least two pathologic test results were required for poor outcome prediction. These results suggest that GWR is a useful additional parameter that may increase the level of certainty for poor outcome prediction in comatose patients after cardiac arrest.

### GWR cut-off value and timing of CCT

Our cut-off value of 1.16 is similar to previously published values. Torbey et al. found a GWR < 1.18 to be 100% specific of poor outcome [11]. The authors obtained GWR at the basal ganglia level in normothermic patients. None of the eleven patients with GWR < 1.18 survived. In the largest cohort so far by Metter et al., a GWR < 1.15 was found to be 100% specific for poor outcome [17]. CCT was obtained within 24 hours and the majority of patients with GWR < 1.15 had been treated with hypothermia. Metter et al. concluded that a GWR < 1.2 had the best overall predictive value, but this threshold misclassified two patients. A of GWR < 1.2 in our patient cohort would have misclassified six patients as having poor outcome. Hence, our data suggest that a threshold of 1.2 can be associated with a substantial number of erroneous poor outcome predictions.

In the study by Metter et al. the number of patients who survived with little or no neurological deficit was small [17]. This outcome distribution may have decreased the likelihood of detecting rare cases that survive with good neurological outcome despite a low GWR. In our study, the proportion of patients surviving with little or no neurological deficit was higher (38%, n = 37).

Inamasu et al. reported a high sensitivity and specificity of the ‘loss of boundary’ (LOB) sign and ‘sulcal effacement’ sign obtained prospectively very early after cardiac arrest [16]. Only one of 52 patients with LOB sign and none of 20 patients with sulcal effacement sign survived with good outcome. These data corroborate the high specificity of early CT signs for poor outcome prediction.

Wu et al. investigated a semi-automated procedure, which used co-registration of CT to a brain atlas and determination of Hounsfield units for several brain regions [15]. In a large cohort, the authors demonstrate that whole brain Hounsfield units obtained from early CCT (most within 24 hours) improved the predictive accuracy of a clinical examination. In this study, only 12% of patients had received hypothermia treatment, and therefore results should be interpreted with care for this patient group.

The majority of CCTs in our study were performed on admission, frequently within a few hours after cardiac arrest. Metter et al. did not find a significant association of attenuation with time since cardiac arrest within a narrow time frame of 24 hours [17]. The distribution of GWR over time in our study, however, suggests that GWR may decrease over the days following cardiac arrest for patients with severe hypoxic-ischemic encephalopathy (Figure 2). Similarly, Wu et al. found a significant decrease in the density of the putamen for CT performed within 24–72 hours as compared to within the first 24 hours after cardiac arrest [15]. No difference was found for other regions, but only few CTs (4%) were performed after day two, hence the power to detect a difference was low.

In our study, sensitivity of poor outcome detection by GWR < 1.16 was 23% for CCT performed within six hours, but increased to 60% for CCT performed between six hours and seven days. This finding, however, may be biased by patient selection and should be interpreted with care. Nonetheless our data argue for a prospective study investigating the prognostic value of GWR beyond six hours after cardiac arrest.

Since many CTs in our study were performed within the first few hours following admission, it is unlikely that hypothermia had substantially influenced the GWR in this subset of patients. However, as hypothermia changes the course of pathophysiological events leading to definite tissue damage in the days after cardiac arrest, it is still possible that hypothermia influences the relationship between early GWR and neurological outcome. Survival with good outcome may be possible in hypothermia patients despite a more severe early injury. Hence, re-evaluation of the prognostic value of GWR for hypothermia patients is mandatory, before GWR can be integrated into pathways for management of these patients.

### Comparison with other outcome parameters

The lower sensitivity of GWR < 1.16 (35% as compared to 53% for NSE and 55% SSEP) and the fact that the vast majority of patients with GWR < 1.16 had either high NSE levels or bilateral absent SSEP may suggest that GWR < 1.16 identifies patients with more severe hypoxic-ischemic encephalopathy than NSE or SSEP. Most of the CCTs in our study were performed within a few hours, but SSEP and NSE were obtained around day three after cardiac arrest. The rather low sensitivity of GWR for the entire study cohort could therefore also be explained by the early time point. Indeed, our data suggest that the sensitivity of GWR increases substantially if CCT is performed > 24 h after admission.

If one accepts a prediction approach that uses a single parameter for poor outcome prediction, the combination of NSE and SSEP increases the sensitivity for detection of poor outcome. Apparently, a relevant number of patients with poor outcome are detected by NSE but not by SSEP and vice versa. If NSE and SSEP are performed, GWR < 1.16 did not significantly increase the number of patients for which poor outcome was detected. However, if one prefers a prediction approach that requires a second confirmatory parameter, GWR has an additional predictive value.

### Limitations of our study

Due to the retrospective design of our study, we could only investigate the subset of patients who had received CCT (n = 111, i.e. 31% of all cardiac arrest patients). CCT was generally ordered by treating physicians if the cause of the cardiac arrest was deemed uncertain or if intracranial complications were suspected. Our results should therefore be extended to the entire cardiac arrest population with care and corroboration by a prospective study avoiding selection bias is desirable. The same caveat applies to the comparison of different prognostic parameters, which could only be performed in the subset of patients who had received NSE, SSEP and CCT.

The results of NSE, SEP and CCT were available to the treating physicians and influenced decisions on limitation of treatment. Therefore, we cannot exclude the possibility of a self-fulfilling prophecy. GWR was calculated for this study and not known to treating physicians. However, reduced GWR reflects brain edema and treatment decisions were influenced by CT if brain edema was reported by the radiologist. As a number of recent studies have indicated limited reliability of prognostic parameters in hypothermia patients, treatment withdrawal within the first days after cardiac arrest is restricted to patients with confirmed brain death at our institution. In the remainder, treatment is continued up to day seven [19]. Hence, a major effect of a self-fulfilling prophecy from treatment withdrawal within the first days after cardiac arrest is unlikely in our study. Nonetheless, unexpected late recovery may occur in individual patients [5], and we cannot fully rule out that treatment withdrawal based on over-interpretation of prognostic parameters has prevented the detection of such exceptional cases.

## Conclusion

Our study confirms the strong association of a low GWR with poor outcome. The optimal cut-off value of GWR was 1.16 in our study, which predicted poor outcome with 100% specificity and 38% sensitivity in our cohort of hypothermia treated patients. Most patients with GWR < 1.16 had absent SSEP or NSE > 97 μg/L. Hence, GWR did not substantially increase the sensitivity to predict poor outcome if these two parameters were available. However, GWR may be useful in a multi-parameter approach and may increase the certainty with which poor outcome can be predicted early after cardiac arrest in patients treated with hypothermia. Further prospective studies are needed to corroborate GWR thresholds and to evaluate the optimal timing of CCT for outcome prediction in cardiac arrest survivors.

## Competing interests

All authors declare no conflict of interests.

## Authors’ contributions

MS carried out the ROI measurements and statistical analysis and drafted the manuscript. CS designed the study, coordinated the collection of the data of the patients and drafted the manuscript. AG carried out the ROI measurements, participated in the statistical analysis and revised the manuscript. JN + FL collected the data of the patients and revised the manuscript. CP gave conceptual advice and revised the manuscript. CL designed the study, reviewed SSEPs and drafted the manuscript. All authors read and approved the final manuscript.

## Supplementary Material

Additional file 1: Figure S1 ROC analysis for different GWR calculation methods. Click here for file
