# Supplementary figures and images for: The prognostic value of gray-white-matter ratio in cardiac arrest patients treated with hypothermia
Source: Scand J Trauma Resusc Emerg Med. 2013 Apr 8;21:23. doi: 10.1186/1757-7241-21-23 (PMC3636054; doi:10.1186/1757-7241-21-23)

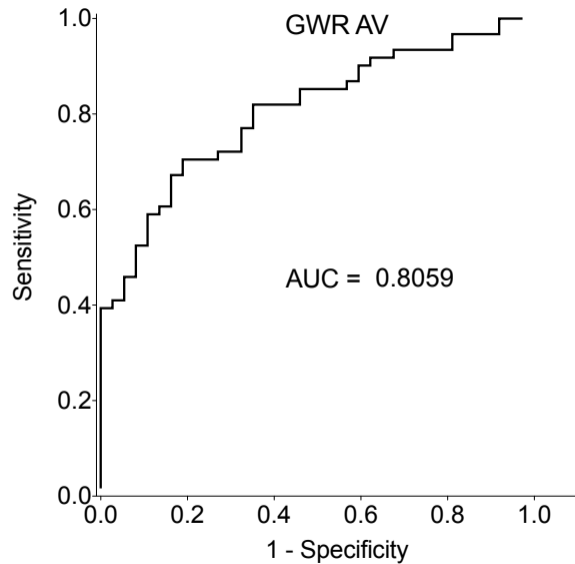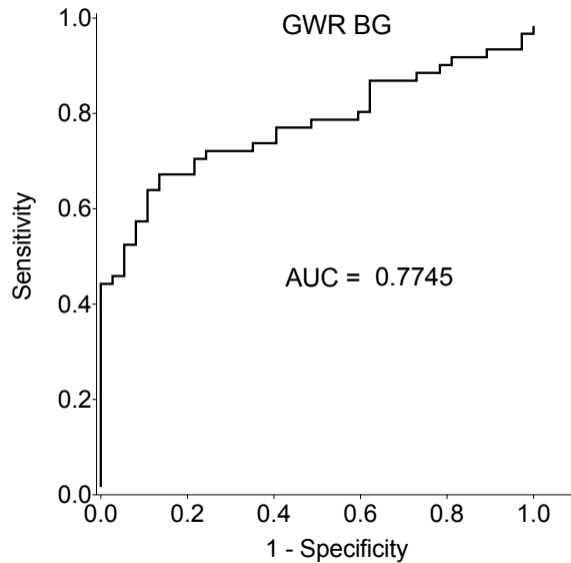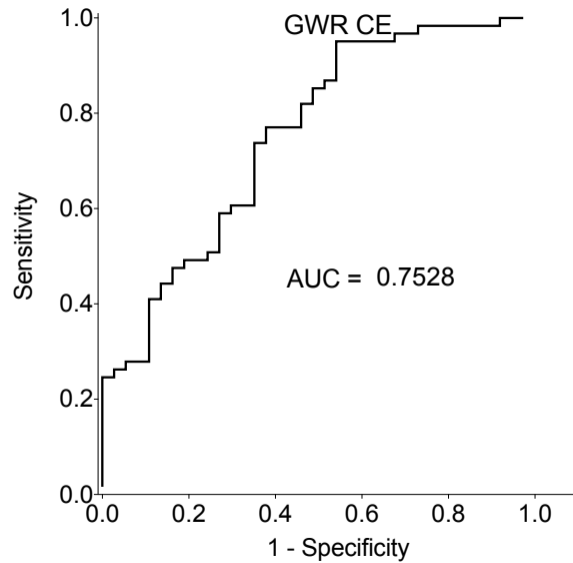

Supplement: Additional file 1: Figure S1 — ROC analysis for different GWR calculation methods. [file 1757-7241-21-23-S1.pdf]
